# Effectiveness and safety of artesunate–amodiaquine versus artemether–lumefantrine for home-based treatment of uncomplicated *Plasmodium falciparum* malaria among children 6–120 months in Yaoundé, Cameroon: a randomized trial

**DOI:** 10.1186/s12879-022-07101-2

**Published:** 2022-02-21

**Authors:** Peter Thelma Ngwa Niba, Akindeh Mbuh Nji, Innocent Mbulli Ali, Lawrence Fonyonga Akam, Cedric Hermann Dongmo, Jean Paul Kengne Chedjou, Calvino Tah Fomboh, William Dorian Nana, Ornella Laetitia Ayem Oben, Abdel Aziz Selly-Ngaloumo, Marcel N. Moyeh, Jude Achidi Ngu, Ambassa Jean Ludovic, Pierre Martiniel Aboh, Marie Carine Enyegue Ambani, Pierrette Albertine Mbarga Omgba, Grâce Bissohong Kotcholi, Linus Moye Adzemye, Danielle Regine Abenkou Nna, Adèle Douanla, Ze Ango, Marie Sophie Ewane, Joel Tewara Ticha, Fritz Mbuh Tatah, Golwa Dinza, Valentine Nchafor Ndikum, Dorothy A. Fosah, Jude D. Bigoga, Michael Alifrangis, Wilfred F. Mbacham

**Affiliations:** 1 MARCAD-DELTAS Program, Laboratory for Public Health Research Biotechnologies, University of Yaoundé I, Yaoundé, Cameroon; 2 The Biotechnology Center, University of Yaoundé I, Yaoundé, Cameroon; 3 Department of Biochemistry, Faculty of Science, University of Yaoundé I, Yaoundé, Cameroon; 4 Department of Biochemistry, Faculty of Science, University of Dschang, Dschang, Cameroon; 5 Department of Biochemistry, Faculty of Medicine and Biomedical Sciences, University of Yaoundé I, Yaoundé, Cameroon; 6 Department of Biochemistry and Molecular Biology, Faculty of Science, University of Buea, Buea, Cameroon; 7 District Medical Center, Minkoa-Meyos, Yaoundé, Cameroon; 8 District Hospital, Ntui, Center Region Cameroon; 9 District Hospital, Cité Verte, Yaoundé, Cameroon; 10 Jamot Hospital, Yaoundé, Cameroon; 11 Department of Pharmacology and African Traditional Medicine, Faculty of Medicine and Biomedical Sciences, University of Yaoundé I, Yaoundé, Cameroon; 12 National Malaria Control Program, Ministry of Public Health, Yaoundé, Cameroon; 13 Centre for Medical Parasitology, Department of Immunology and Microbiology, Faculty of Health and Medical Sciences, University of Copenhagen, Copenhagen, Denmark; 14 Department of Infectious Diseases, Copenhagen University Hospital, Copenhagen, Denmark; 15 Cameroon Coalition Against Malaria, P.O. Box 8094, Yaoundé, Cameroon

**Keywords:** *Plasmodium falciparum*, Malaria, Effectiveness, Safety, Artesunate**-**amodiaquine, Artemether**-**lumefantrine, Cameroon

## Abstract

**Background:**

Many studies have reported high efficacy and safety of artesunate**-**amodiaquine (AS**-**AQ) and artemether**-**lumefantrine (AL) when administered under direct observation in Cameroon. There is paucity of data to support their continuous use in home**-**based treatment of uncomplicated *Plasmodium falciparum* malaria in Cameroon. Hence, this study aimed to assess the effectiveness and safety of AS**-**AQ versus AL for home**-**based treatment of uncomplicated *P*. *falciparum* malaria among children 6**–**120 months in Yaoundé, Cameroon.

**Methods:**

A two**-**arm, open**-**label, randomized, controlled trial comparing the equivalence of AS**-**AQ (experimental group) and AL (control group) was carried out from May 2019 to April 2020 at two secondary hospitals in Yaoundé. Participants were randomized to receive either AS**-**AQ or AL. After the first dose, antimalarial drugs were given at home, rather than under direct observation by a study staff. The conventional on**-**treatment and post**-**treatment laboratory and clinical evaluations were not done until day 3 of the full antimalarial treatment course. The evaluation of effectiveness was mainly based on per protocol polymerase chain reaction adjusted adequate clinical and parasitological response (PP PCR adjusted ACPR) on day 28 post**-**treatment. Safety was based on assessment of adverse events (AEs) and severe adverse events (SAEs) from day 1 to day 28.

**Results:**

A total of 242 children were randomized to receive AS**-**AQ (n = 114) and AL (n = 128). The PP PCR adjusted day 28 cure rates were [AS**-**AQ = 96.9% (95% CI, 91.2**–**99.4) versus AL = 95.5% (95% CI, 89.9**–**98.5), P = 0.797]. Expected mild to moderate adverse events were reported in both arms [AS**-**AQ = 83 (84.7%) versus AL = 99 (86.1%), P = 0.774]. The most common adverse events included: transient changes of hematologic indices and fever.

**Conclusions:**

This study demonstrated that AS**-**AQ and AL are effective and safe for home management of malaria in Yaoundé. The evidence from this study supports the parallel use of the two drugs in routine practice. However, the findings from this study do not describe the likely duration of antimalarial effectiveness in holoendemic areas where multiple courses of treatment might be required.

*Trial registration:* This study is a randomized controlled trial and it was retrospectively registered on 23/09/2020 at ClinicalTrials.gov with registration number NCT04565184.

**Supplementary Information:**

The online version contains supplementary material available at 10.1186/s12879-022-07101-2.

## Background

Malaria remains a major public health challenge in sub**-**Saharan Africa, Southeast Asia and Latin America despite the deployment and scaling up of control measures [1]. In 2020, the disease accounted for 241 million cases and 627,000 related deaths worldwide [1]. Since April 2001, the World Health Organization (WHO) has recommended the swift change of policy from monotherapies to artemisinin**-**based combination therapies (ACTs) in the treatment of uncomplicated *P*. *falciparum* malaria due to rapid emergence and dispersal of drug resistant parasites [2]. Clinical trials are the mainstay for monitoring the efficacy of ACTs in vivo [3]. WHO has approved six ACTs: artesunate**-**amodiaquine (AS**-**AQ), artemether**-**lumefantrine (AL), dihydroartemisinin**-**piperaquine (DHA**-**PPQ), artesunate**-**mefloquine (AS**-**MQ), artesunate + sulfadoxine**-**pyrimethamine (AS + SP) and artesunate**-**pyronaridine (AS**-**PY) [4, 5]. The sixth ACT (AS**-**PY) was recently adopted due to its high efficacy and safety profile despite initial concerns raised in previous studies [6, 7]. Cameroon adopted the use of AS**-**AQ and AL in 2004 and 2006 respectively for the treatment of *P*. *falciparum* malaria [8]. AS**-**AQ and AL have been in continuous use since their introduction in 2004 and 2006. Both AS**-**AQ and AL are used in the Southern regions, but in the North and Far North regions, AL is primarily used for treatment and sulfadoxine**-**pyrimethamine (SP**-**AQ) is used for seasonal malaria prophylaxis among children 3 to 59 months [9].

Antimalarial drug effectiveness is a composite assessment that encompasses clinical efficacy (the performance of the medicine under controlled conditions) and real**-**world clinical practice (less optimal conditions) [10]. Several ACTs have been shown to be highly efficacious [11–15] and effective [16, 17] in the treatment of malaria in different endemic countries across the world. The ACTs already adopted by the WHO are safe with no major adverse events reported [18–21].

Presently, the ACTs are under threat due to the identification of single nucleotide polymorphisms (SNPs) in the *P*. *falciparum* kelch 13 propeller gene conferring resistance to the artemisinins [22]. The prevalence of the mutations (F446I, N458Y, N458Y, Y493H, R539T, I543T, P553L, R561H, P574L, C580Y) has been well documented in the Greater Mekong sub**-**region in Southeast Asia [23–25]. The occurrence of the C469Y [26], R561H [27–29], P574L [27–29] and A675V [26] polymorphisms has also been recently reported in Africa. This may negatively affect the gains already achieved in the drive towards malaria elimination in endemic countries if measures to curb the threat are not urgently put in place. Furthermore, it has been shown that the emergence of drug resistant parasites could be delayed by using multiple first line ACTs [30, 31].

The WHO recommends the change of treatment lines if the efficacy of ACTs is less than the minimum benchmark of 90%. Many studies have reported high efficacy and safety of artesunate**-**amodiaquine (AS**-**AQ) and artemether**-**lumefantrine (AL) under strict drug administration in Cameroon [13, 32, 33]. There is paucity of data to support their continuous use in home**-**based treatment of uncomplicated *P*. *falciparum* malaria Cameroon. Hence, this study aimed to assess the effectiveness and safety of AS**-**AQ versus AL for home**-**based treatment of uncomplicated *P*. *falciparum* malaria among children 6–120 months in Yaoundé, Cameroon.

## Methods

### Study sites

The study was coordinated at the District Hospital, Cité Verte and District Medical Center, Minkoa**-**Meyos located in the Health Districts of Cité Verte and Nkolbisson, respectively in Yaoundé, Cameroon. The town of Yaoundé (3°50′N 11°31′E) in the Center Region is found at an elevation of 760 m (2493 feet) above sea level [34]. The average temperature is 23.8 °C while the annual rainfall is 1628.3 mm [34]. It has four distinct seasons: two rainy seasons (March–May/June, September–November) and two dry seasons (December–February, June/July–August). Malaria transmission is holoendemic with peak transmission taking place during and immediately following the two rainy seasons with an entomological inoculation rate that varies from 0–90.0 infective bites per person per year [35].

### Study design

This study was a two**-**arm, equivalence, open**-**label, randomized controlled trial comparing the effectiveness and safety of AS**-**AQ and AL among children age 6 to 120 months over a 28 days period of follow**-**up. The experimental group was AS**-**AQ while the control group was AL.

### Study period

The study was conducted from May 2019 to April 2020. This corresponded with the period during which the first participant was enrolled and the last child successfully followed**-**up.

### Sample size determination

The data regarding the efficacy of the two ACTs were derived from previous studies conducted in Yaoundé. The cure rates were 96.8% for AS**-**AQ in 2014 [12] and 100% for AL in 2009 [32]. The sample size for equivalence was determined using the WHO acceptable cure rate of 95% [3] for AS**-**AQ within 95% CI, a power of 90% and assuming a difference of 10% between the AS**-**AQ and AL. The following sample size determination formula was employed [36, 37]:

$$n = 2 \times \left[ {\left( {Z_{{1 - {\alpha \mathord{\left/ {\vphantom {\alpha 2}} \right. \kern-\nulldelimiterspace} 2}}} + Z_{1 - \beta } } \right)^{2} \times p{{\left( {1 - p} \right)} \mathord{\left/ {\vphantom {{\left( {1 - p} \right)} {\left( d \right)^{2} }}} \right. \kern-\nulldelimiterspace} {\left( d \right)^{2} }}} \right]$$

where: n = sample size; Z_α/2_ = Z value at two**-**tailed alpha (0.05) at 95% CI = 1.96, Z_β_ = Z value of 1**-**β = 1.28, P_1_ = proportion of successes on AS**-**AQ = 95.0%; d = difference between the 2 drugs

$${\text{n}} = { 2} \times \left[ {\left( {{1}.{96 } + { 1}.{28}} \right)^{{2}} \left( {0.{95}} \right)\left( {0.0{5}} \right)/\left( {0.{1}} \right)^{{2}} } \right] = { 99}.{73}$$

A minimum sample of 100 patients was required for the study. With a 14% increase to allow for loss to follow**-**up and withdrawals during the 28**-**day follow**-**up period, 114 patients were allocated to each of the two study arms. Considering a non**-**compliance rate of 14% to the treatment guideline due to the number of doses (6 doses) administered and timing of doses, 14 additional participants were allocated to the AL arm giving a total of 128.

### Study procedures

The study procedure was nearly identical to the standard WHO 28**-**day efficacy protocol [3]. The main exceptions were that only the first doses of antimalarial drugs were supervised (by clinical staff) and there was no planned clinical or laboratory evaluation until day 3.

### Screening, eligibility and enrolment

Patients who fulfilled all the following criteria were included in the study: (i) Children of either gender, aged 6 months to 120 months were recruited; (ii) Uncomplicated *P. falciparum* malaria confirmed by microscopy using Giemsa**-**stained thick film with an asexual parasite density within the range > 0 to $\le$ 200,000 parasites/μl with a slight modification of the WHO recommended guideline [3]; (iii) Presenting with fever (axillary temperature ≥ 37.5 °C) or having a history of fever in the preceding 24 h; (iv) Able to ingest tablets orally (either suspended in water or uncrushed with food); (v) Willingness to participate in the study with written assent from parent/guardian; (vi) Willing and able to attend the clinic on stipulated regular follow**-**up visits.

The final decision for enrollment depended on the parents or guardians willingness to supervise home**-**based treatment and to facilitate post**-**treatment clinical and laboratory evaluations. The parents and guardians always gave written consent in addition to written assent for their children to participate in the study.

Patients who presented with any one of the following criteria were excluded from participating in the study**:** (i) Mixed or mono**-**infection with another *Plasmodium* species detected by microscopy; (ii) Children who were currently suffering or had the following within the last 2 months: tuberculosis, HIV, schistosomiasis, diabetes mellitus, cardiovascular disease, gout, rheumatoid arthritis, underlying chronic hepatic or renal disease, hypoglycemia, jaundice, respiratory distress, and other inflammatory related diseases. (iii) Signs/symptoms indicating severe/complicated malaria according to WHO criteria (WHO definition) such as: parasitemia ≥ 5% of red blood cell count, not able to drink or breast feed, persistent vomiting (> 2 episodes within previous 24 h), convulsions (> 1 episode within previous 24 h), lethargic/unconscious, severe anemia (hemoglobin < 5 g/dl) and axillary temperature > 40 ºC; (iv) Serious gastrointestinal disease; v) Presence of severe malnutrition defined as a child aged between 6**–**60 months whose weight**-**for**-**height is below –3 z**-**score (W/H < 70%) or has symmetrical edema involving at least the feet or has a mid**-**upper arm circumference < 115 mm; (vi) Regular medication which may interfere with antimalarial pharmacokinetics; (vii) History of hypersensitivity reactions or contraindications to any of the medicine (s) being tested or used as alternative treatment (s); (viii) Individuals who took part in antimalarial efficacy and safety studies 3 months before the start of the study; (ix) Participants who took antimalarial drugs in the past one month.

### Randomization of study participants

A randomization list was produced according to a randomization allocation schedule generated by a computer**-**based randomization program in blocks of 20 s [38]. The allocation of participants was concealed in opaque envelopes that were opened sequentially by the study pharmacist once assent was provided and enrollment validated by the study physician. The children were randomized to receive either AS**-**AQ or AL. The randomization codes were recorded on the case report forms against the study identification numbers.

### Trial drug administration and follow-up

Artesunate**-**amodiaquine (AS**-**AQ, Winthrop, Sanofi, Paris, France, lot numbers: M0001501**-**01, M0001502**-**01, M0001503**-**01) oral tablets supplied were fixed**-**dosed combinations and available in three dosage forms. Each prescription was based on age groups and weights. The drug, AS**-**AQ was administered for three days (Additional file 1).

Artemether**-**lumefantrine (AL, Dispersible Coartem®: Novartis, Basel, Switzerland, lot numbers: KX673, KT866) oral tablets were also fixed**-**dose combinations provided in blister packs of different doses. Each tablet contained 20 mg artemether and 120 mg lumefantrine. The prescription was based on the weight of the child in line with the manufacturer’s recommended guidelines. The drug, AL was given to each participant for three days (Additional file 1).

A replacement dose was given to any child if vomiting occurred less than 30 min after ingestion. The participant was given half the dose if vomiting took place after 30 min of ingestion. Participants who had more than one episode of vomiting within one hour after drug intake were excluded from the study.

Drug intake was supervised by study staff for the first dose; subsequent doses were supervised by participants’ parents or guardians. Parents/guardians of participants were advised on the time and mode of administration for the 3 days treatment unobserved at home. Antipyretic medications such as paracetamol syrup or tablets were administered together with the study drugs.

Follow**-**up visits were carried out on days 3, 7, 14, 21, 28 or any other day that the child felt unwell in order to evaluate clinical and parasitological resolution of their malaria episodes as well as adverse drug events. Community health workers were also used to locate the houses of the study participants and to ensure compliance with follow**-**up schedules.

The rescue treatments administered to participants who developed severe malaria during follow**-**up included: injectable artesunate, injectable artemether and quinine infusion. These treatments were administered in line with the WHO 2015 guidelines [38].

### Clinical evaluation

A standard physical examination was done for body weight, axillary temperature, blood pressure (systolic/diastolic), respiratory rate, and pulse rate (heart rate) at baseline (day 0 before dosing) and on days 3, 7, 14, 21, 28 and during unscheduled visits.

### Laboratory analyses

#### Detection and quantification of the malaria parasite

The presence of malaria parasite was detected in capillary blood or venous blood using the SD BIOLINE Malaria Ag *P*. *falciparum* (HRP2/pLDH) (Standard Diagnostics, Incorporation, South Korea) and CareStart™ Malaria HRP2/pLDH (Pf/PAN) Combo (Access Bio, Incorporation, Somerset, New Jersey, United State of America) rapid diagnostic test kits before proceeding with microscopy. Thick and thin blood films for parasite counts and speciation of non**-***falciparum* species were obtained and examined at screening on day 0 to confirm adherence to the inclusion and exclusion criteria [39]. Thick blood films were also examined on days 3, 7, 14, 21, and 28 or on any other day if the patient returned spontaneously and parasitological reassessment was required. The slide for each patient was prepared in duplicates. Thick and thin blood films were dried and stained with 10% Giemsa for at least 15**–**20 min. Giemsa**-**stained thick and thin blood films were examined at a magnification of 1000 × to identify the parasite species and to determine the parasite density. At least 200 white blood cells (WBCs) were counted. Parasite density was calculated by multiplying the number of parasites counted per microscopic field by a factor of 40 on the assumption that the average count of WBC is 8000 per µl. If on counting 200 WBCs, the number of parasites was not up to 100, the count continued till 500 WBCs and calculations made to get the estimated parasitemia of the patient. Parasitemia was reported in parasites/μl. A microscopy slide was considered negative when examination of 1000 white blood cells or 100 fields containing at least 10 white blood cells per field revealed no asexual parasites. The presence of gametocytes on an enrollment or follow**-**up slide was also recorded. To detect the presence of gametocyte, at least 1000 white blood cells were counted. Two qualified microscopists read all the slides independently, and parasite densities were calculated by averaging the two counts. Blood smears with discordant results (differences between the two microscopists in species diagnosis, in parasite density of > 50% or in the presence of parasites) were re**-**examined by a third, independent microscopist, and parasite density was calculated by averaging the two closest counts. Additionally, 10% of all the slides were randomly selected and read by a senior microscopist for quality control. Enrollment into the study depended on a positive malaria rapid diagnostic test and microscopy.

#### Measurement of hematology and biochemistry parameters

Hemoglobin level was measured from finger prick blood sample using portable Hemocue 201 and Hemocue 301 spectrophotometers (HemoCue®, Ängelholm, Sweden). Whole blood was collected in ethylene diamine tetra acetic acid (EDTA) tubes and dry tubes for hematology and biochemistry analyses respectively. The samples in EDTA tubes were properly mixed to avoid blood clot before laboratory analyses. Hematological parameters were analyzed with URIT 3000 hematology analyzer (URIT Medical Electronic Co., Limited, Guangxi, China) and Mindray 3000 Plus (Shenzen Mindray Bio**-**medical Electronic Co., Limited, Shenzen, China). Blood biochemistry parameters were analyzed using the URIT 810 semi**-**automated Chemistry analyzer (URIT Medical Electronic Co., Limited, Guangxi, China). Measurements were carried out on days 0, 7, 14, 28 and during unplanned visits. Quality control was carried out daily to validate each test run. Each parameter was evaluated by comparing it values with the established reference ranges (Additional file 2).

#### Malaria parasite DNA extraction

The malaria parasite deoxyribonucleic acid (DNA) was extracted from whole blood collected in EDTA tubes and dried blood spots (DBS) on Whatman® N^o^ 3 mm filter papers using the EZNA Biotek method according to the manufacturer’s guidelines. DNA was extracted from the whole blood in EDTA tubes/DBS on day 0 (before treatment) and during recurrence of parasitemia on day 7 onwards (cases of treatment failure).

#### Genotyping of malaria parasites to differentiate recrudescence from reinfection

Genotyping was done in order to differentiate a recrudescence (same parasite strain) from a newly acquired infection (different parasite strain). The *P*. *falciparum* merozoite surface protein 1 (*Pfmsp***-**1), *P*. *falciparum* merozoite surface protein 2 (*Pfmsp***-**2) and *P*. *falciparum* glutamate**-**rich protein (*Pfglurp*) genes were used to discriminate reinfections from recrudescence as previously described [40, 41]. Summarily, DNA fragments obtained from amplification of baseline samples (day 0) and on the day of recurrent parasitemia were compared according to band size and number, considering the 3 families of *Pfmsp***-**1 (MAD20, RO33, K1), the 2 families of *Pfmsp***-**2 (FC27, 3D7/IC) and single family type of *Pfglurp*. Cases were categorized as new infections when there were no common bands between day 0 and the day of recurrent parasitemia. However, when there was at least one common band between baseline sample and that of the day of parasite reappearance for any of the 3 markers (even if there were additional bands on day 0) the case was recrudescence. Malaria parasite infected cases were considered not to be clinical failures if their recurrent parasitemia were classified as new infections rather than recrudescent infections. The gene amplification was done using the Biometra T3 Thermocycler (United Kingdom).

#### Assessment of caregivers’ compliance to treatment schedules

Adherence to prescribed treatment schedules was assessed through interviews (self**-**reporting) on day 3 post**-**treatment about the timing of treatment, the number of doses administered and the number of days over which drug was given. The interviews were conducted at the health facility. Used packs of drugs were inspected where available. Participants who had persistent vomiting (vomited more than once within one hour) after the first dose was administered, were enrolled without meeting all the inclusion criteria**,** withdrew consent after day 0 and did not show up on day 3 post**-**treatment were excluded from the assessment. Adherence was classified as follows: poor adherence (adherence rate < 95%) and good or perfect adherence (adherence rate ≥ 95%).

#### Evaluation of effectiveness

Treatment effectiveness was evaluated based on clinical and parasitological outcomes. This assessment was done according to the WHO 2009 guideline for monitoring therapeutic efficacy studies [3]. Based on this guideline, treatment outcome was classified as treatment failure and treatment success. Treatment failure is defined as early treatment failure (ETF), late clinical failure (LCF) and late parasitological failure (LPF). Treatment success is defined as adequate clinical and parasitological response (ACPR).

#### Assessment of safety

Safety was based on assessment of adverse events (AEs) and severe adverse events (SAEs) on day 1 to day 28. A physician from the study team was designated to monitor the safety of ACTs. Adverse events were recorded through interviews or self**-**reporting about previous symptoms and about symptoms that have emerged since the previous follow**-**up visit. A clinical examination was performed to determine any adverse event. In addition to clinical assessment, hematological parameters, liver function and renal function were evaluated for abnormal values. An adverse event (AE) is defined according to the International Conference on Harmonization guidelines as any untoward medical occurrences in a patient administered a pharmaceutical product and which does not necessarily have a causal relationship with the treatment of interest [42]. A severe adverse event (SAE) is considered as an untoward medical occurrence that at any dose results in death, is life threatening, requires or prolongs hospitalization, results in persistent and significant disability, is a congenital anomaly/birth defect or is another medically important condition [42].

### Study outcomes

1. The primary outcome was the proportion of patients with PP PCR**-**corrected cure rates for AS**-**AQ in comparison with AL after a follow**-**up period of 28 days.
2. The secondary outcome was proportion of participants with adverse events and severe adverse events for AS**-**AQ in comparison with AL after a follow**-**up period of 28 days.
3. The other exploratory outcomes investigated were: i) The proportion of patients with PP PCR**-**corrected cure rates for AS**-**AQ in comparison with AL on day 7 and day14. ii) The number of children in the two drug groups with parasitemia on day 3, fever on day 3 and gametocyte carriage on days 0, 3, 7, 14, 21 and/or 28. iii) The proportion of children who adhered to AS-AQ and AL treatment guidelines. iv) The evolution of biological parameters between day 0 and day 7.

### Data management

The Microsoft Excel 2010 (Microsoft Corporation, Redmond, Washington, United States of America) was used to design the data extraction sheet. Data was double entered by two independent data clerks. Discrepancies were resolved by mutual consent after discussion and independent review from the third data clerk. This was done in order to improve on the quality and acceptability of data generated. The database in Microsoft Excel was piloted and validated before completion of the process.

### Statistical analysis

The International Business Machines Statistical Software Package for Social Sciences (IBM SPSS) version 20.0 software package (IBM Corporation, Armonk, New York, United States of America) was used for data analysis. The Pearson chi**-**square test (χ^2^) or Fisher’s exact test was used to determine the association between qualitative variables depending on the percentage of cells having an expected frequency < 5. The Shapiro**–**Wilk test was use to check the normality of quantitative variables. The mean differences for quantitative variables were estimated using the Student’s t test for independent samples if they complied with the normal distribution hypothesis and the Mann**–**Whitney U test with median when non**-**normally distributed. Normal data was represented as mean $\pm$ standard deviation while non**-**normal data was summarized as median (interquartile range).

The effectiveness of AS**-**AQ vs. AL was assessed based on unadjusted and adjusted PCR ACPR. The following parameters were used: intention**-**to**-**treat (ITT) population, per**-**protocol (PP) population and Kaplan**–**Meier (K**-**M) survival estimates. The significance of observed differences between the treatment arms based on ITT and PP analyses were assessed using the Pearson chi**-**square test or Fisher’s exact test. The log rank (Mantel**-**Cox) chi**-**square test was used to investigate the difference between the drug groups based on K**-**M survival estimates. The significance of observed differences between the safety parameters of AS**-**AQ and AL were also evaluated using the Pearson chi**-**square test or Fisher’s exact test.

The assessment of equivalence of the two drug groups was based on: P < 0.05 (not equivalent) or P > 0.05 (equivalent).

All P**-**values were two**-**tailed, and values less than 0.05 were considered to be statistically significant at 95% confidence interval.

### Ethical considerations

This study was reviewed and approved by the Center Regional Ethics Committee (CE Nº 05859/CRERSHC/2019) and National Ethics Committee (Nº 2018/07/1091/CE/CNERSH/SP) for Human Health Research, Ministry of Public Health, Yaoundé, Cameroon. Administrative authorizations were also sought from the Director of District Hospital Cité Verte, Director of District Medical Center Minkoa**-**Meyos, Center Regional Delegate of Public Health and the Minister of Public Health. Parents/guardians of participants were explained the full scope of the study and the right of their children to participate or not without any prejudice; participation could be stopped at any time without any explanation. Written consent and assent in French or English were obtained from all parents or guardians of eligible children before enrollment into the study. Subjects with malaria were treated in accordance with the National Malaria Control Program guideline. The clinical trial was registered retrospectively and posted at ClinicalTrials.gov on 23/09/2020 with registration number: NCT04565184 (https://clinicaltrials.gov/ct2/show/NCT04565184).

## Results

### Trial profile on enrollment, allocation, intervention, follow-up and data analysis

Out of 987 participants screened for eligibility, 242 met the inclusion criteria and were enrolled into the study while 745 children who did not meet the inclusion criteria were excluded from the study. A total of 242 participants were randomized to receive either artesunate**-**amodiaquine (AS**-**AQ) or artemether**-**lumefantrine (AL) in the ratio of 114:128. In the AS**-**AQ group, 16 children were excluded due to protocol violation (n = 6) and failure to appear on day 3/other days of follow**-**up (n = 10). Similarly, in the AL group, 13 children were excluded because of the following reasons: withdrawal of consent (n = 1), protocol violation (n = 3) and failure to appear on day 3/other days of follow**-**up (n = 8).

**Fig. 1:**
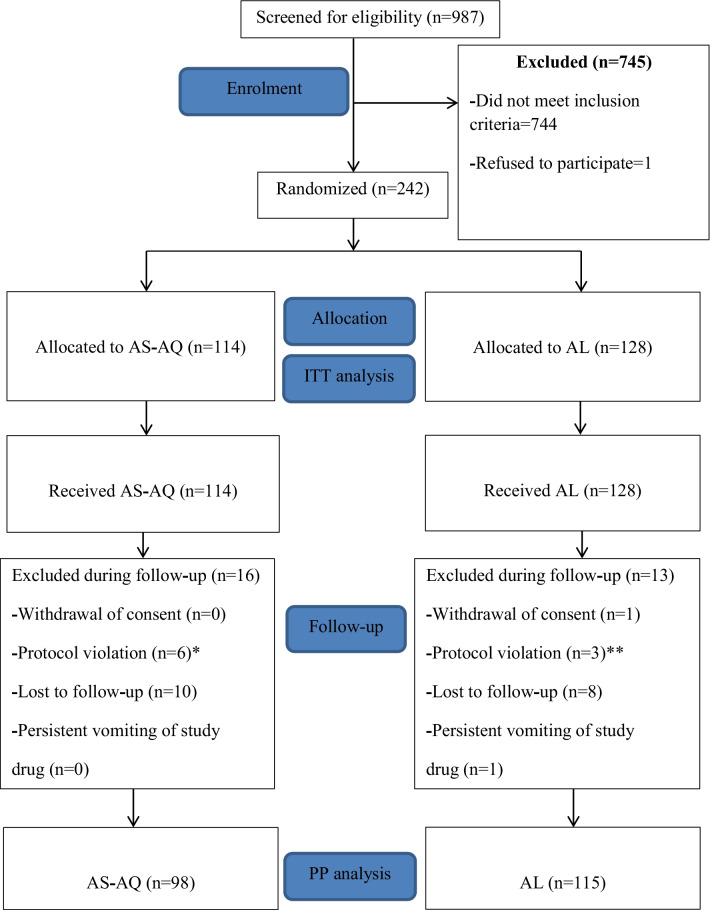
Trial profile of effectiveness and safety study on artesunate-amodiaquine (AS-AQ) and artemether-lumefantrine (AL) in Yaoundé, Cameroon. *Enrollment violation (1 participant had baseline parasitaemia > 200,000 parasites/µl, 2 had only positive results by malaria rapid diagnostic test and 2 had signs of severe malaria); Treatment violation (1 did not comply with the treatment procedure)**.** **Enrollment violation (1 participant had baseline parasitaemia > 200,000 parasites/µl and 1 had only positive result by malaria rapid diagnostic test); Treatment violation (1 did not comply with the treatment procedure)

In the AS**-**AQ arm, 114 participants were included in the intention**-**to**-**treat (ITT) analysis while 98 children were included in the per protocol (PP) analysis after the exclusion of 16 children. Likewise, in the AL arm, 128 participants were included in the intention**-**to**-**treat (ITT) analysis while 115 children were included in the per protocol (PP) analysis after the exclusion of 14 children. The 28 days period of follow**-**up was adopted in both treatment arms (Fig. 1).

### Baseline characteristics of the study participants treated with artesunate-amodiaquine (AS-AQ) and artemether-lumefantrine (AL) using the ITT population

Out of 114 participants who received AS**-**AQ, there were more males than females (68:46). Likewise, out of 128 children assigned to the AL arm, there were more males than females (72:56). In both drug arms, majority of the study participants were enrolled in the age group 60**–**120 months [AS**-**AQ 58 (50.9%) versus AL 73 (57.0%)]. The median age was 58.5 months in the AS**-**AQ arm while in the AL arm it was 64.5 months.

Furthermore, there were no statistically significant differences observed in all the categorical and quantitative variables evaluated in the two comparable independent treatment arms (P > 0.05). This permitted the comparison between the two drug groups at baseline (Table 1).

**Table 1:** Baseline characteristics of the study participants treated with AS-AQ and AL using the ITT population

Characteristic/Category	AS-AQ (n = 114, %)	AL (n = 128, %)	P-value*
Gender ratio (Male: Female)	46:68	56:72	0.593
Age group (months)			
< 60	56 (49.1)	55 (43.0)	0.338
60**–**120	58 (50.9)	73 (57.0)	
Median (IQR)	58.5 (50)	64.5 (54)	0.660
Range (min, max)	(6**–**120)	(8**–**120)	
Heart rate (beats per minute)			
Median (IQR)	100 (15)	100 (18)	0.839
Range (min, max)	(24**–**164)	(40**–**150)	
Systolic blood pressure (mm/Hg)			
Median (IQR)	100 (10)	100 (10)	0.476
Range (min, max)	(70**–**157)	(70**–**158)	
Diastolic blood pressure (mm/Hg)			
Median (IQR)	60 (10)	60 (10)	0.657
Range (min, max)	(40**–**90)	(36**–**140)	
Respiratory rate (breaths per minute)			
Median (IQR)	30 (7)	30 (9)	0.411
Range (min, max)	(14**–**78)	(16**–**60)	
Body weight (Kg)			
Median (IQR)	19.0 (9.1)	20.0 (8.8)	0.902
Range (min, max)	(7.7**–**33.0)	(6.9**–**48.0)	
Axillary temperature (°C)			
Median (IQR)	37.2 (0.8)	37.4 (1.1)	0.982
Range (min, max)	(35.7**–**40.0)	(35.6**–**40.2)	
Hemoglobin concentration (g/dl)			
Mean $\pm$ SD	10.4 $\pm$ 1.7	10.6 $\pm$ 1.6	0.196
Range (min, max)	(5.6**–**13.6)	(5.8**–**15.4)	
Parasitemia (parasites/µl)			
GMPD	3940	4259	0.736
Range (min, max)	(79**–**331,131)	(79**–**575,440)	
Total bilirubin (mg/dl)			
Median (IQR)	0.0478 (0.0851)	0.0657 (0.0968)	0.150
Range (min, max)	(0.0006**–**0.7640)	(0.0016**–**0.6100)	
Aspartate aminotransferase (U/L)			
Median (IQR)	42.5100 (27.0700)	40.1845 (18.0330)	0.237
Range (min, max)	(11.8870**–**173.6800)	(1.9759**–**141.5700)	
Alanine aminotransferase (U/L)			
Median (IQR)	21.5400 (14.2800)	22.0220 (10.5990)	0.754
Range (min, max)	(3.8954**–**109.0800)	(3.6267**–**371.9800)	
Creatinine (mg/dl)			
Median (IQR)	0.4858 (0.2059)	0.5130 (0.2211)	0.566
Range (min, max)	(0.1558**–**10.6600)	(0.1845**–**0.9954)	

*n*  Number of participants enrolled into the AS**-**AQ and AL drug arms on day 0, *SD * Standard deviation, *GMPD* Geometric mean parasite density, *IQR* Interquartile range, *Min* minimum value; *Max *maximum value, *Determined by using Student *t* independent test, Mann**–**Whitney *U* test or Pearson chi**-**square test

### Primary outcome evaluation of in vivo effectiveness of AS-AQ and AL for the treatment of uncomplicated *Plasmodium falciparum* malaria on day 28 (intention-to-treat and per protocol analyses)

The in vivo effectiveness on day 28 using unadjusted PCR intention**-**to**-**treat (ITT) analyses were 82.5% (95% CI, 74.2**–**88.9) for AS**-**AQ and 83.6% (95% CI, 76.0**–**89.6) for AL. The ITT cure rates adjusted by PCR were 83.2% (95% CI, 75.0**–**89.6) for AS**-**AQ and 85.6% (95% CI, 78.2**–**91.2) for AL. Conversely, the PCR unadjusted in vivo effectiveness obtained from per protocol (PP) analyses were 95.9% (95% CI, 89.9**–**98.9) for AS**-**AQ and 93.0% (95% CI, 86.8**–**97.0) for AL. The PCR adjusted ACPR derived from PP analyses were 96.9% (95% CI, 91.2**–**99.4) for AS**-**AQ and 95.5% (95% CI, 89.9**–**98.5) for AL. The ITT and PP (PCR uncorrected and corrected) cure rates were not significantly different across arms (P > 0.05) (Table 2).

**Table 2:** Primary outcome evaluation of in vivo effectiveness of AS-AQ and AL for the treatment of uncomplicated *Plasmodium falciparum* malaria day 28 (ITT and PP analyses)

Effectiveness evaluation	AS-AQ (95% CI)	AL (95% CI)	P-value
ITT analysis without PCR correction	n = 114	n = 128	0.644
ETF	3	4	
LCF	1	2	
LPF	0	2	
ACPR	94 (82.5%, 74.2–88.9)	107 (83.6%, 76.0–89.6)	
Persistent vomiting of study drug, LTFU, WT and PV	16	13	
PP analysis without PCR correction	n = 98	n = 115	0.720
ETF	3	4	
LCF	1	2	
LPF	0	2	
ACPR	94 (95.9%, 89.8–98.9)	107 (93.0%, 86.8–97.0)	
ITT analysis with PCR correction	n = 114	n = 128	0.685
ETF	3	4	
LCF	0	0	
LPF	0	1	
ACPR	94 (83.2%, 75.0–89.6)	107 (85.6%, 78.2–91.2)	
Persistent vomiting of study drug, LTFU, WT and PV	16	13	
Reinfection	1	3	
PP analysis with PCR correction	n = 97	n = 112	0.797
ETF	3	4	
LCF	0	0	
LPF	0	1	
ACPR	94 (96.9%, 91.2–99.4)	107 (95.5%, 89.9–98.5)	
Reinfection	1	3	

*ITT* Intention**-**to**-**treat, *PP* Per protocol, *n*  Number of participants enrolled on day 0 or followed**-**up until day 28, *AS****-****AQ*  Artesunate**-**amodiaquine, *AL * Artemether**-**lumefantrine, *ETF*  Early treatment failure, *LCF * Late clinical failure, *LPF*  Late parasitological failure, *ACPR * Adequate clinical and parasitological response, *LTFU*  Lost to follow**-**up, *WT*  Withdrawn, *PV*  Protocol violation, *CI*  Confidence interval at 95%, In the PP analysis with PCR correction, the re**-**infected cases were not used to determine ACPR

Treatment failures for the two drugs were due to early treatment failure (ETF), late clinical failure (LCF) and late parasitological failure (LPF) unadjusted and adjusted by PCR. There were 8 cases of treatment failures (3 for AS**-**AQ and 5 for AL) when adjusted by PCR. Recrudescence accounted for only one case of LPF in the AL arm after PCR correction. There were four cases of reinfections (1 for AS**-**AQ arm and 3 for AL arm) registered during the period of follow**-**up (Table 2).

### Assessment of in vivo effectiveness on D7 and D14 based on per-protocol analysis

The adjusted PCR adequate clinical and parasitological response (Adjusted PCR ACPR) rates on day 7 and day 14 were: 96.9% for AS**-**AQ and 96.5% for AL. The cure rates did not differ when compared on day 7 and/or day 14 for either arm (P = 1.000) (Table 3).

**Table 3:** Assessment of PCR-adjusted therapeutic responses on D7 and D14 (per protocol analysis)

Treatment outcome	Day 7		P-value*	Day 14		P-value*
	AS-AQ (%)	AL (%)		AS-AQ (%)	AL (%)	
	n = 98	n = 115		n = 98	n = 115	
ACPR	95 (96.9)	111 (96.5)	1.000	95 (96.9)	111 (96.5)	1.000
ETF	3 (3.1)	4 (3.5)		3 (3.1)	4 (3.5)	
LCF	0 (0.0)	0 (0.0)		0 (0.0)	0 (0.0)	
LPF	0 (0.0)	0 (0.0)		0 (0.0)	0 (0.0)	

*ACPR* Adequate clinical and parasitological response, *ETF* Early treatment failure, *LCF* Late clinical failure, *LPF* Late parasitological failure, *AS****-****AQ* Artesunate-amodiaquine, *AL* Artemether**-**lumefantrine, *n* Number of participants followed**-**up until day 7 or day 14; n values for each row are cumulative (that is, the row 2 n values for ETF do not reflect any new ETFs since day 7, just the cumulative number of ETFs thus far), *Determined using Fisher’s exact test for categorical variables

### Kaplan–Meier survival estimates for in vivo effectiveness of AS-AQ versus AL (PP without and with PCR correction)

The Kaplan**–**Meier survival cure rates without PCR correction were 96.5% (95% CI, 93.2**–**99.8) for AS**-**AQ and 93.8% (95% CI, 89.1**–**98.5) for AL. The two groups were not statistically different when compared (P = 0.363) (Table 4 and Additional file 3).

**Table 4:** Kaplan–Meier survival estimates for in vivo effectiveness of AS-AQ versus AL (PP without and with PCR correction)

Effectiveness evaluation	AS-AQ (95% CI)	AL (95% CI)	P-value*
	n = 114	n = 128	
PP without PCR correction			
ACPR day 28	94	107	0.363
Censored (Persistent vomiting of study drug, LTFU, WT and PV)	16	13	
Treatment failures	4	8	
Cumulative incidence of success, day 28	96.5% (93.2–99.8)	93.8% (89.1–98.5)	
PP with PCR correction			0.605
ACPR day 28	94	107	
Censored (Persistent vomiting of study drug, LTFU, WT,PV and re-infections)	17	16	
Treatment failure (recrudescence)	3	5	
Cumulative success rate, day 28	97.4% (94.1–100.0)	96.1% (92.4–99.8)	

*PP* Per protocol, *AS****-****AQ* Artesunate**-**amodiaquine, *AL* Artemether**-**lumefantrine, *LTFU* Lost to follow**-**up, *WT * Withdrawn, *PV* Protocol violation, *n* Number of participants enrolled on day 0 or followed**-**up until day 28, *CI* Confidence interval, *Determined using the Log rank (Mantel**-**Cox) chi**-**square test

The Kaplan**–**Meier survival cure rates with PCR correction were 97.4% (95% CI, 94.1**–**100.0) for AS**-**AQ and 96.1% (95% CI, 92.4**–**99.8) for AL. Similarly, the two groups were not statistically different when compared (P = 0.605) (Table 4 and Additional file 3).

### Evaluation of fever, parasitemia clearance and adherence to treatment guideline on D3 of the study

It was observed that 93 (92.1%) of study participants in the AS**-**AQ arm and 99 (86.1%) in the AL arm of study participants did not have fever on day 3 (P = 0.162). The proportion of children who cleared their parasites on day 3 were 70 (69.3%) for AS**-**AQ and 85 (73.9%) for AL (P = 0.453) (Table 5).

**Table 5:** Proportion of study participants with fever, parasitemia and adherence on D3 of study

Characteristic/category	AS-AQ (%)	AL (%)	P-value*
	n = 101	n = 115	
Proportion of fever on D3			
Yes	8 (7.9)	16 (13.9)	0.162
No	93 (92.1)	99 (86.1)	
Proportion of parasitemia on day 3			
Yes	31 (30.7)	30 (26.1)	0.453
No	70 (69.3)	85 (73.9)	
Adherence to treatment guideline			
Yes	100 (99.0)	115 (99.1)	1.000
No	1 (1.0)	1 (0.9)	

*n*  Sample size, *Determined using Pearson chi**-**square test or Fisher’s exact test for categorical variables, Only participants who came for the day 3 visit post**-**treatment were included in the analyses, Participants were excluded from analyses if they vomited the study drug more than once, violated enrollment criteria, were lost**-**to**-**follow**-**up or withdrew from the study before day 3

Good adherence to treatment guidelines were recorded in the AS**-**AQ [100/101 (adherence rate = 99.0%, 95% CI, 94.6**–**100.0)] and AL [115/116 (adherence rate = 99.1%, 95% CI, 95.3**–**100.0)] drug arms. The adherence rate was not statistically different between the two treatment groups [P = 1.000] (Table 5).

### Proportion of gametocyte carriage at enrollment and post-treatment

The total number of the study participants carrying gametocytes on day 0, 3, 7, 14, 21 and/or 28 by microscopy were 5 (4.5%) for AS**-**AQ and 5 (4.0%) for AL (P = 1.000). In the AS**-**AQ arm, the highest proportion of gametocyte (n = 2, 2.0%) was recorded on day 3 and day 7 of follow-up while the lowest proportion was documented on day 0 (n = 1, 0.9%). In contrast, in the AL arm the highest prevalence of gametocyte (n = 2, 1.6%) was registered on day 0 while the lowest rate of (n = 1, 0.9%) was recorded on day 3, day 7, and day 14. There was complete gametocyte clearance on day 21 and 28 in both drug arms. None of the study participants in the two drug arms had more than one episode of gametocyte carriage post**-**treatment.

### Safety and tolerability endpoints in the study arms of AS-AQ versus AL from day 1 to day 28 (per protocol analysis)

A total of 83 (84.7%) participants in the AS**-**AQ arm and 99 (86.1%) participants in the AL arm had mild to moderate adverse events from day 1 to day 28 based on per protocol analysis. Most of the study participants in both drug arms had at least two or more adverse events due to multiple responses. Out of 44 adverse events reported, the most common were: leucocytosis [AS**-**AQ = 14 (14.3%) versus AL = 20 (17.4%), P = 0.537], lymphocytosis percentage [AS**-**AQ = 19 (19.4%) versus AL = 16 (13.9%), P = 0.283], granulocytopenia percentage [AS**-**AQ = 20 (20.4%) versus AL = 22 (19.1%), P = 0.815], lymphocytosis number [AS**-**AQ = 20 (20.4%) versus AL = 17 (14.8%), P = 0.280], thrombocytosis [AS**-**AQ = 27 (27.6%) versus AL = 38 (33.0%), P = 0.386] and fever [AS**-**AQ = 17 (16.3%) versus AL = 28 (24.3), P = 0.150].

Only two types of adverse events had a statistically significant difference between the 2 drug arms: eosinophilia + basophilia**-**% [AS**-**AQ = 17 (17.3%) versus AL = 7 (6.1%), P = 0.010] and low diastolic blood pressure [AS**-**AQ = 0 (0.0%) versus AL = 6 (5.2%), P = 0.032]. All the adverse events resolved post**-**treatment (Table 6).

**Table 6:** Frequency of adverse drug events (mild to moderate WHO grading) in the AS-AQ and AL drug arms from day 1 to day 28 (PP)

Adverse event	AS-AQ (%)	AL (%)	P-value*
	n = 98	n = 115	
Using PP populations			
Gastrointestinal tract			
Vomiting	0 (0.0)	1 (0.9)	1.000
Nausea	0 (0.0)	1 (0.9)	1.000
Anorexia	3 (3.1)	4 (3.5)	1.000
Abdominal pain	6 (6.1)	2 (1.7)	0.147
Diarrhea	1 (1.0)	3 (2.6)	0.626
Neuropsychiatric			
Headache	2 (2.0)	1 (0.9)	0.595
Difficulty in hearing	0 (0.0)	1 (0.9)	1.000
Blood circulatory system			
Leucopenia	2 (2.0)	2 (1.7)	1.000
Leucocytosis	14 (14.3)	20 (17.4)	0.537
Lymphocytosis (%)	19 (19.4)	16 (13.9)	0.283
Eosinophilia + basophilia (%)	17 (17.3)	7 (6.1)	0.010**
Granulocytopenia (%)	20 (20.4)	22 (19.1)	0.815
Lymphocytosis (number)	20 (20.4)	17 (14.8)	0.280
Eosinophilia + basophilia (number)	9 (9.2)	6 (5.2)	0.259
Granulocytopenia (number)	3 (3.1)	3 (2.6)	1.000
Granulocytosis (number)	1 (1.0)	3 (2.6)	0.626
Erythropenia	2 (2.0)	2 (1.7)	1.000
Erythrocytosis	5 (5.1)	8 (7.0)	0.573
Low hemoglobin level (anemia)	9 (9.2)	10 (8.7)	0.901
Low hematocrit	13 (13.3)	10 (8.7)	0.284
Decrease in mean cell volume	5 (5.1)	5 (4.3)	1.000
Decrease in mean cell hemoglobin	5 (5.1)	9 (7.8)	0.424
Decrease in mean cell hemoglobin concentration	5 (5.1)	8 (7.0)	0.573
Thrombocytopenia	2 (2.0)	3 (2.6)	1.000
Thrombocytosis	27 (27.6)	38 (33.0)	0.386
Liver function			
Increase in aspartate aminotransferase	7 (7.1)	13 (11.3)	0.299
Increase in alanine aminotransferase	7 (7.1)	9 (7.8)	0.805
Kidney function			
Increase in creatinine	0 (0.0)	1 (0.9)	1.000
Cardiovascular system			
Decrease in heart rate	1 (1.0)	2 (1.7)	1.000
Low systolic blood pressure	0 (0.0)	1 (0.9)	1.000
High systolic blood pressure	2 (2.0)	6 (5.2)	0.292
Low diastolic blood pressure	0 (0.0)	6 (5.2)	0.032**
High diastolic blood pressure	4 (4.1)	3 (2.6)	0.706
Respiratory system			
Increase in respiratory rate	1 (1.0)	3 (2.6)	0.626
Difficulty in breathing	2 (2.0)	0 (0.0)	0.211
Others			
Fever	16 (16.3)	28 (24.3)	0.150
Asthenia	1 (1.0)	3 (2.6)	0.626
Catarrh	1 (1.0)	1 (0.9)	1.000
Cough	3 (3.1)	10 (8.7)	0.087
Splenomegaly	0 (0.0)	1 (0.9)	1.000
Abnormal chest	1 (1.0)	0 (0.0)	0.460
Skin rash	0 (0.0)	4 (3.5)	0.126
Perioral dermatitis	0 (0.0)	1 (0.9)	1.000
Rhinorrhea	0 (0.0)	1 (0.9)	1.000
Overall prevalence	83 (84.7)	99 (86.1)	0.774

Increase for clinical, hematological and biochemical parameters is defined as any value above the normal range; Decrease for clinical, hematological and biochemical parameters is defined as any value below the normal range; *Determined by using Pearson’s chi**-**square test or Fisher’s exact test; **P < 0.05**-**Statiscally significant

### Evolution of biological parameters among study participants treated with AS-AQ and AL (Day 0 and Day 7) (PP)

A total of 22 parameters were evaluated in the 2 drug arms between day 0 and day 7. Majority of these parameters 13 (59.0%) did not significantly change over time. However, 9 biological parameters had a significant change between day 0 and day 7. A total of 53 (58.9%) and 66 (63.5%) of participants had abnormal values in the AS**-**AQ and AL groups respectively. Out of the 9 parameters, the following had significant increase between day 0 and day 7: white blood cell count**-**WBC (× 10^9^/L) [median (IQR)] [AS**-**AQ, day 0 = 7.75 (6.20) versus day 7 = 9.75 (5.70**)**, P = 0.006; AL, day 0 = 8.50 (5.1) versus day 7 = 9.30 (5.5), P = 0.029] and platelet count**-**PLT (× 10^9^/L) [median (IQR)] [AS**-**AQ, day 0 = 247 (158) versus day 7 = 336 (226), P < 0.0001, AL, day 0 = 198 (157) versus day 7 = 334 (197), P < 0.0001] (Additional file 4).

## Discussion

The present study had as primary objective to determine the effectiveness and safety of artesunate**-**amodiaquine (AS**-**AQ) versus artemether**-**lumefantrine (AL) for a period of 28 days among children infected with uncomplicated *P*. *falciparum* malaria in Yaoundé, Cameroon. The effectiveness of the two drugs was assessed under less optimal conditions to mimic what happens in the communities 17 years after their adoption and implementation in Cameroon. The approach adopted for this study is a modification of the standard procedure recommended by WHO for monitoring the efficacy of ACTs [3]. The standard method describes what is expected in health care but not what is observed.

The evaluation of in vivo effectiveness using per protocol (PP) analysis gave an adjusted PCR**-**ACPR value above the WHO benchmark of 90% for AS**-**AQ. Similar adjusted PCR cure rates for AS**-**AQ have been recorded before in Cameroon [12] and Burkina Faso [15]. The adjusted PCR cure rate for AS**-**AQ is higher than the range of 60.0% to 91.7% obtained from studies conducted at the slope of Mount Cameroon [43] and other countries [16, 44–48]. Conversely, the adjusted PCR**-**ACPR of the present study is lower than range of 98.1%**-**100.0% registered in supervised clinical trials conducted in Cameroon [13], Burkina Faso [14, 15], Ghana [49] and Senegal [50]. The assessment of in vivo effectiveness using PP analysis also registered a corrected PCR cure rate above 90% for AL. This finding is in agreement with the adjusted PCR cure rate of 95.1% registered in rural Tanzania in 2011 [17]. The adjusted PCR**-**ACPR is lower than the rates of 96.0–100.0% reported in malaria endemic countries in sub**-**Saharan Africa [13, 49–51], Southeast Asia [11] and South America [52]. A systematic review and meta**-**analysis on the therapeutic efficacy of AL (Coartem®) for the treatment of *P. falciparum* malaria in Africa gave a pooled PCR adjusted 97.0% [53]. The adjusted PCR cure rate for AL is higher than the range of 77.7–94.0% previously documented in several antimalarial drug effectiveness [16, 44, 46, 48] and efficacy [15] studies conducted elsewhere. The lower limit of the confidence interval for AL success, as reported, was less than 90% (the WHO benchmark), while the lower limit of the confidence interval for AS**-**AQ was slightly above this benchmark (91.2%). The differences observed in the effectiveness rates of AS**-**AQ and AL may be due to heterogeneity of study settings, participants, malaria transmission patterns, sample sizes, and development of resistance against artemisinin partner drugs. Even though, mutations conferring resistance to the artemisinins have not been reported in Cameroon, there is a need to regularly monitor the effectiveness and efficacy of antimalarial drugs as recommended by the WHO.

The comparison of in vivo effectiveness on day 28 did not show any significant difference between AS**-**AQ and AL. This is in agreement with the findings of the comparative effectiveness and efficacy studies in which the two drugs were used [13, 44, 48]. The insignificant difference in the in vivo effectiveness of AS**-**AQ versus AL disagrees with the results reported in Burkina Faso [15, 46]. These findings confirm the parallel use of AS**-**AQ and AL for the treatment of uncomplicated *P. falciparum* malaria.

Furthermore, the present study registered very few cases of reinfection 14 days post**-**treatment. This observation tallies with those of other studies that showed an increase in the effectiveness [16, 43, 44, 46, 48, 54] and efficacy [11–15] of AS**-**AQ and AL after correction with PCR. The AL drug arm documented more cases of reinfections after 14 day of follow**-**ups when compared with the AS**-**AQ drug arm [16, 44, 46, 48, 54]. The lack of direct supervision during drug administration did not seem to lower the effectiveness of the ACTs below the acceptable WHO benchmark of 90%. This could be due to perfect adherence by the caregivers to the dosing regimens. There are conflicting claims on the impact of adherence on antimalarial drug efficacy and effectiveness. Some studies did not find a difference in the PCR cure rates between ACT dispensed supervised and unsupervised [17, 45, 54]. This is inconsistent with the findings from another study that reported a significant difference between supervised and unsupervised therapeutic responses [55]. The difference observed may be due insufficient patient adherence. It has been revealed that antimalarial drug efficacy and effectiveness is influenced by a plethora of factors. These factors include: delayed access to drugs, bioavailability, drug quality, drug storage conditions, age [17, 56, 57], multiplicity of infection [58, 59], malaria parasite density [56, 60], immunity [61], pharmacogenomics [62, 63] and nutrition [64].

In both drug arms, fever and parasite disappeared gradually by day 3. Rapid fever and parasite clearance rates have been reported before in Senegal/Ivory Coast [65, 66], Cameroon [13], and Togo [67]. The presence of gametocytes at enrollment and during follow**-**up days was found to be higher in the AS**-**AQ group when compared to the AL group. These observations are in harmony with those reported in Senegal/Ivory coast [65, 66] and Togo [67]. A meta**-**analysis from individual patient data conducted by the Worldwide Antimalarial Resistance Network (WWARN) Gametocyte Study Group confirmed that AL is more effective than the AS**-**AQ fixed dose combination in preventing gametocyte carriage shortly after treatment [68]. The authors suggested that the non**-**artemisinin partner drugs and the timing of artemisinin dosing are important determinants responsible for post**-**treatment gametocyte dynamics [68]. Information on gametocyte carriage reported during this study is important to guide Cameroon government’s policy on malaria control and elimination especially in regions where transmission occur throughout the year.

In addition, expected mild to moderated adverse events were documented in the two drug arms that resolved during follow**-**up. There was no severe adverse event registered among the study participants. Previous pharmacovigilance studies on AS**-**AQ and AL have mostly reported the presence of common adverse events associated with the gastrointestinal tract and neuropsychiatric systems [13, 18, 19]. This approach of monitoring adverse events relies on self**-**reporting by the patients, parents or guardians. A major setback of this method is recall bias. Some parents or guardians may actually have over**-**reported perceived adverse events because of anxiety about the possibility of adverse events.

The study drugs had a statistical significant effect on 9 biological parameters on day 0 and day 7 in the AS**-**AQ and AL arms. It was also shown that AS**-**AQ and AL drugs had varying effects on alanine aminotransferase (ALAT) and creatinine (CREA) levels on day 0 and day 7. These results corroborate with those realized in Senegal and Ivory Coast [65]. These observations also confirm the findings of the study on the efficacy and safety of ACTs in Garoua and Mutengene, Cameroon reported by Nji and colleagues in 2015 [13]. The resolution of abnormal values during follow**-**up is an indication that AS**-**AQ and AL are well tolerated and safe.

## Strengths and limitations of the study

The major strengths of this study are: (1) This work filled significant gaps in knowledge about the performance of AS**-**AQ and AL in Cameroon, which should provide important support for policy**-**making. (2) Both regimens were studied concurrently in the town of Yaoundé in Cameroon seventeen years after policy implementation. The existence of data from previous efficacy trials of the same regimens in the same region was also important. (3) The fact that the study was performed in a holoendemic region in Cameroon is actually important, because the national malaria control program needs data from diverse transmission settings in order to devise rational policy. (4) The design was intended to "mimic the routine standard of care" by omitting clinical supervision of treatment, clinical evaluation, and laboratory testing after day zero and before day three. This is actually a strength given the study team's emphasis on effectiveness.

The major limitations of this study are: (1) There was no direct clinical observation of antimalarial drug dosing (after dose 1), and standard clinical and laboratory evaluations were not conducted after day zero and before day 3. (2) The study was conducted in urban and peri**-**urban settings of Yaoundé. These areas are characterized with holoendemic malaria transmission. This may not present the reality of what happens in the other malaria transmission settings in Cameroon and globally. (3) The estimated sample size may have been inadequate to establish the equivalence of the two drugs. (4) Families who were willing to be adherent to medication and clinical/laboratory evaluation schedules may not have been representative of the entire population from which the subjects were drawn. Hence, study results may not be generalizable.

## Conclusions

This study demonstrated that AS**-**AQ and AL are effective and well tolerated for home management of uncomplicated *P*. *falciparum* malaria among children in Yaoundé, Cameroon. The PP cure rates adjusted by PCR and safety parameters for the two drugs were not statistically different and are said to be substantively the same when compared. The two regimens seem to have retained their effectiveness and safety profiles for over 17 years, in spite of frequent administration likely driven by the intensity of malaria transmission. The evidence from this study supports the government’s policy on the parallel use of AS**-**AQ and AL in routine practice in the Southern regions of Cameroon. However, the findings from this study do not describe the likely duration of antimalarial effectiveness in holoendemic areas where multiple courses of treatment might be required. It was also reported that the lower limit of confidence interval was slightly below or above the WHO threshold of 90% for AL and AS**-**AQ respectively. Thus, there is a need to continuously monitor the effectiveness and safety profile of AS**-**AQ and AL in Cameroon.

## Supplementary Information

**Additional file 1:** Study treatment

**Additional file 2: **Reference ranges of heart rate, systolic blood pressure, diastolic blood pressure, respiratory rate, hematology parameters and biochemistry parameters

**Additional file 3: **Kaplan**–**Meier survival curve for the effectiveness of AS**-**AQ versus AL (PP without and with PCR correction)

**Additional file 4: **Evolution of biological parameters among study participants treated with AS**-**AQ and AL (Day 0 and Day 7) (PP)

## Data Availability

The datasets used and/or analyzed during the current study are available from the corresponding author on reasonable request.

## Abbreviations

ACT: Artemisinin**-**based combination therapy
ACPR: Adequate clinical and parasitological response
AL: Artemether**-**lumefantrine
ALT: Alanine aminotransferase
AS**-**AQ: Artesunate**-**amodiaquine
AST: Aspartate aminotransferase
CREA: Creatinine
ETF: Early treatment failure
LCF: Late clinical failure
LPF: Late parasitological failure
WHO: World Health Organization
WWARN: Worldwide Antimalarial Resistance Network
