# Supplementary material for: Effectiveness and safety of artesunate–amodiaquine versus artemether–lumefantrine for home-based treatment of uncomplicated Plasmodium falciparum malaria among children 6–120 months in Yaoundé, Cameroon: a randomized trial
Source: BMC Infect Dis. 2022 Feb 21;22:166. doi: 10.1186/s12879-022-07101-2 (PMC8862275; doi:10.1186/s12879-022-07101-2)
Supplement: Supplementary file 1 — Additional file 1: Study treatment [file 12879_2022_7101_MOESM1_ESM.docx]

**Additional file 1: Study treatment**

**Table 1 Artesunate**-**Amodiaquine (ASAQ)**

| **Treatment** | **Weight Range (Kg)** | **Day 0** | | **Day 1** | | **Day 2** | |
| --- | --- | --- | --- | --- | --- | --- | --- |
|  |  |  | |  | |  | |
|  |  | **0 hour** | **12 hours** | **24 hours** | **36 hours** | **48 hours** | **60 hours** |
| (25 mg/67.5 mg  blister of 3 tablets) | 4.5 kg**-**8 kg | 1 tablet | NA | 1 tablet | NA | 1 tablet | NA |
| (50 mg/135 mg  blister of 3 tablets) | 9 kg**-**17 kg | 1 tablet | NA | 1 tablet | NA | 1 tablet | NA |
| (100 mg/270 mg  blister of 3 tablets) | 18 kg**-**35 kg | 1 tablet | NA | 1 tablet | NA | 1 tablet | NA |

*NA: Not applicable*

**Table 2 Artemether + Lumefantrine (AL)**

| **Treatment** | | **Weight Range (Kg)** | **Day 0** | | **Day 1** | | **Day 2** | |
| --- | --- | --- | --- | --- | --- | --- | --- | --- |
|  |  |  |  | |  | |  | |
|  |  |  | **0 hour** | **8 hours** | **24 hours** | **36 hours** | **48 hours** | **60 hours** |
| 20 mg/120 mg  (blister of 6 tablets) | | 5 kg – <15 kg | 1 tablet | 1 tablet | 1 tablet | 1 tablet | 1 tablet | 1 tablet |
| 20 mg/120 mg  (blister of 12 tablets) | | 15 kg – <25 kg | 2 tablets | 2 tablets | 2 tablets | 2 tablets | 2 tablets | 2 tablets |
| 20 mg/120 mg  (blister of 18 tablets) | | 25 kg – <35 kg | 3 tablets | 3 tablets | 3 tablets | 3 tablets | 3 tablets | 3 tablets |
|  | |  |  |  |  |  |  |  |
|  | | | | | | | | |
