# Supplementary material for: Effectiveness and safety of artesunate–amodiaquine versus artemether–lumefantrine for home-based treatment of uncomplicated Plasmodium falciparum malaria among children 6–120 months in Yaoundé, Cameroon: a randomized trial
Source: BMC Infect Dis. 2022 Feb 21;22:166. doi: 10.1186/s12879-022-07101-2 (PMC8862275; doi:10.1186/s12879-022-07101-2)
Supplement: Supplementary file 2 — Additional file 2: Reference ranges of heart rate, systolic blood pressure, diastolic blood pressure, respiratory rate, hematology parameters and biochemistry parameters [file 12879_2022_7101_MOESM2_ESM.docx]

**Additional file 2: Reference ranges of heart rate, systolic blood pressure, diastolic blood pressure, respiratory rate, hematology parameters and biochemistry parameters**

**Table 1 Clinical Parameters**

| **Vital sign** | **Infant** | **Child** | **Pre-Teen/Teen** |
| --- | --- | --- | --- |
|  | **0 to 12 months** | **1 to 11 years** | **12 years and up** |
| Heart rate | 100 to 160 beats per minute (bpm) | 70 to 120 bpm | 60 to 100 bpm |
| Respiration rate (breaths) | 0 to 6 months  30 to 60 breaths per minute (bpm)  6 to 12 months  24 to 30 bpm | 1 to 5 years  20 to 30 (bpm)  6 to 11 years  12 to 20 bpm | 12 to 18 bpm |
| Blood pressure ([systolic](https://www.mottchildren.org/health-library/sts15384#sts15384-sec)/[diastolic](https://www.mottchildren.org/health-library/std120739#std120739-sec)) | 0 to 6 months  65 to 90/45 to 65 millimeters of mercury (mm Hg)  6 to 12 months  80 to 100/55 to 65 mm Hg | 90 to 110/55 to 75 mm Hg | 110 to 135/65 to 85 mm Hg |

**Table 2 Hematology Parameters**

| **Parameter** | **Unit** | **Normal Range** |
| --- | --- | --- |
| White blood cell count (WBC) | x10^9^/L | 4.0**-**10.0 |
| Lymphocyte percent (LYM %) | % | 20.0**-**40.0 |
| Basophil+eosinophil percent (MID %) | % | 1.0**-**15.0 |
| Granulocyte percent (GRAN %) | % | 50.0**-**70.0 |
| Lymphocyte number (LYM #) | x10^9^/L | 0.6**-**4.1 |
| Basophil+eosinophil number (MID #) | x10^9^/L | 0.1**-**1.8 |
| Granulocyte number (GRAN #) | x10^9^/L | 2.0**-**7.8 |
| Red blood cell count (RBC) | x10^12^/L | 3.5**-**5.5 |
| Hemoglobin (HGB) | g/dl | 11.0**-**15.0 |
| Hematocrit (HCT) | % | 36.0**-**48.0 |
| Mean cell volume (MCV) | fl | 80.0**-**99.0 |
| Mean cell hemoglobin (MCH) | pg | 26.0**-**32.0 |
| Mean cell hemoglobin concentration (MCHC) | g/dl | 32.0**-**36.0 |
| Platelet (PLT) | x10^9^/L | 100**-**300 |

**Table 3 Biochemistry Parameters**

| **Parameter** | **Unit** | **Normal Range** |
| --- | --- | --- |
| Total Bilirubin | mg/dl | Up to 1.00 |
| Aspartate aminotransferase | U/L | Men <38  Women <32 |
| Alanine aminotransferase | U/L | Men <41  Women <31 |
| Creatinine | mg/dl | 0.60 – 1.30 |
