# Supplementary material for: Effectiveness and safety of artesunate–amodiaquine versus artemether–lumefantrine for home-based treatment of uncomplicated Plasmodium falciparum malaria among children 6–120 months in Yaoundé, Cameroon: a randomized trial
Source: BMC Infect Dis. 2022 Feb 21;22:166. doi: 10.1186/s12879-022-07101-2 (PMC8862275; doi:10.1186/s12879-022-07101-2)
Supplement: Supplementary file 3 — Additional file 3: Kaplan–Meier survival curve for the effectiveness of AS-AQ versus AL (PP without and with PCR correction) [file 12879_2022_7101_MOESM3_ESM.docx]

**Additional file 3: Kaplan**-**Meier survival curve for the effectiveness of AS**-**AQ versus AL (PP without and with PCR correction)**


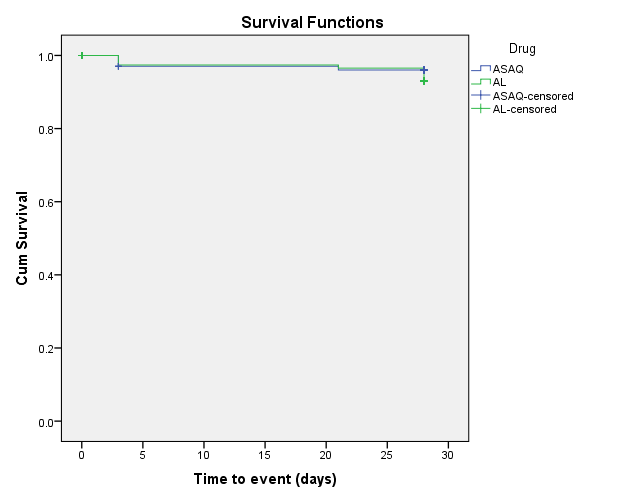


**Fig. 1a Kaplan**-**Meier survival curve for the effectiveness of AS**-**AQ versus AL (PP without PCR correction)**


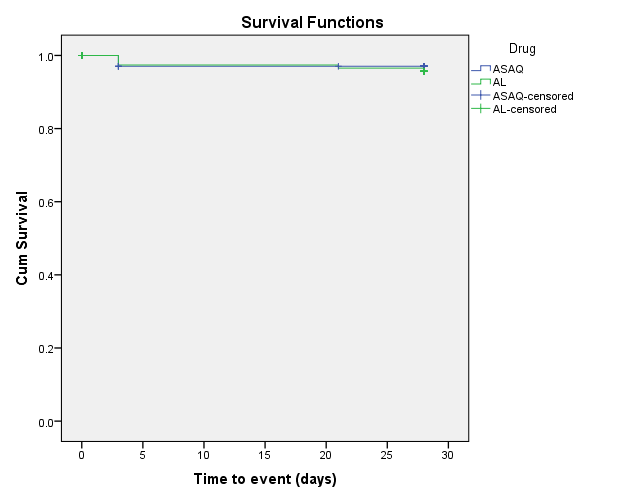


**Fig. 1b Kaplan**-**Meier survival curve for the effectiveness of AS**-**AQ versus AL (PP with PCR correction)**
