# Supplementary material for: Effectiveness and safety of artesunate–amodiaquine versus artemether–lumefantrine for home-based treatment of uncomplicated Plasmodium falciparum malaria among children 6–120 months in Yaoundé, Cameroon: a randomized trial
Source: BMC Infect Dis. 2022 Feb 21;22:166. doi: 10.1186/s12879-022-07101-2 (PMC8862275; doi:10.1186/s12879-022-07101-2)
Supplement: Supplementary file 4 — Additional file 4: Evolution of biological parameters among study participants treated with AS-AQ and AL (Day 0 and Day 7) (PP) [file 12879_2022_7101_MOESM4_ESM.docx]

**Additional file 4: Evolution of biological parameters among study participants treated with ASAQ and AL (Day 0 and Day 7)**

**Table 1 Evolution of biological parameters among study participants treated with ASAQ and AL (Day 0 and Day 7)**

| **Parameter** | **AS**-**AQ** | | **P**-**value** | **AL** | | **P**-**value** |
| --- | --- | --- | --- | --- | --- | --- |
|  | **Day 0** | **Day 7** |  | **Day 0** | **Day 7** |  |
| Heart rate (beats per minute) [median (IQR)] | n=86 | | **0.004*** | n=104 | | **0.002*** |
|  | 100 **(**14) | 100 **(**12) |  | 100 **(**14) | 100 **(**12) |  |
| Systolic blood pressure (mm/Hg**)** [median (IQR)] | n=84 | | 0.594 | n=100 | | 0.502 |
|  | 100 (10) | 100 (10) |  | 100 (10) | 100 (10) |  |
| Diastolic blood pressure (mm/Hg**)** [median (IQR)] | n=85 | | 0.090 | n=100 | | 0.055 |
|  | 60 (10) | 60 (10) |  | 60 (10) | 60 (10) |  |
| Respiratory rate (breaths per minute) [median (IQR)] | n=83 | | 0.264 | n=96 | | 0.865 |
|  | 30 (7) | 30 (7) |  | 30 (8) | 30 (6) |  |
| White blood cell count**-** WBC (x10^9^/L) [median (IQR)] | n=90 | | **0.006*** | n=104 | | **0.029*** |
|  | 7.75 (6.20) | 9.75 (5.70**)** |  | 8.50  (5.1) | 9.30  (5.5) |  |
| Lymphocyte percent**-**LYM % (%) (Mean$\pm$SD)/ [median (IQR)] | n=90 | | **0.018*** | n=104 | | 0.344 |
|  | 42.13$\pm$ 10.29 | 45.64 $\pm$11.61 |  | 45.25 (15.90) | 45.50 (10.60) |  |
| Basophil+eosinophil percent**-**MID % (%) (Mean$\pm$SD) | n=90 | | 0.425 | n=104 | | 0.237 |
|  | 11.93 $\pm$3.58 | 12.30$\pm$ 3.38 |  | 11.59$\pm$ 3.28 | 12.08$\pm$ 2.66 |  |
| Granulocyte percent**-** GRAN % (%) (Mean$\pm$SD)/ [median (IQR)] | n=90 | | **0.011*** | n=104 | | 0.271 |
|  | 45.93 $\pm$12.30 | 41.92$\pm$ 11.40 |  | 41.95 (15.50) | 42.45 (11.40) |  |
| Lymphocyte number**-**LYM # (x10^9^/L) [median (IQR)] | n=90 | | **<0.0001*** | n=104 | | **0.002*** |
|  | 3.20 (2.80) | 4.40 (3.00) |  | 3.20 (2.50) | 3.90  (2.40) |  |
| Basophil+eosinophil number**-**MID # (x10^9^/L)  [median (IQR)] | n=90 | | **0.004*** | n=104 | | **0.006*** |
|  | 0.90 (0.90) | 1.20 (0.90) |  | 0.90 (0.70) | 1.10  (0.90) |  |
| Granulocyte number**-**GRAN # (x10^9^/L)  [median (IQR)] | n=90 | | 0.519 | n=104 | | 0.269 |
|  | 3.80 (2.30) | 4.05 (2.50) |  | 3.70 (2.80) | 4.15 (2.50) |  |
| Red blood cell count**-**RBC (x10^12^/L)  [median (IQR)] | n=90 | | 0.784 | n=104 | | 0.356 |
|  | 4.61 (0.89) | 4.48 (0.86) |  | 4.64 (0.91**)** | 4.59 (0.87) |  |
| Hemoglobin**-**HGB (g/dl) (Mean$\pm$SD) | n=90 | | 0.948 | n=106 | | 0.532 |
|  | 10.46$\pm$ 1.54 | 10.46 $\pm$1.24 |  | 10.75 $\pm$ 1.60 | 10.82 $\pm$1.18 |  |
| Hematocrit**-**HCT (%) (Mean$\pm$SD) | n=90 | | 0.991 | n=104 | | 0.710 |
|  | 38.03 $\pm$7.53 | 37.04$\pm$ 5.96 |  | 37.28$\pm$ 8.76 | 37.61 $\pm$5.55 |  |
| Mean cell volume**-**MCV (fl) (Mean$\pm$SD) | n=90 | | 0.695 | n=104 | | 0.339 |
|  | 81.15$\pm$ 9.39 | 81.39$\pm$ 8.53 |  | 80.93 $\pm$10.61 | 81.79 $\pm$9.42 |  |
| Mean cell hemoglobin**-**MCH (pg) (Mean$\pm$SD) | n=90 | | 0.324 | n=104 | | 0.871 |
|  | 50.03 $\pm$254.13 | 23.42 $\pm$2.89 |  | 23.74  $\pm$ 3.77 | 23.68  $\pm$3.19 |  |
| Mean cell hemoglobin concentration**-**MCHC (g/dl) [median (IQR)] | n=90 | | **0.030*** | n=104 | | 0.479 |
|  | 28.60 (3.20) | 28.80 (3.60) |  | 28.65 (4.1) | 28.80  (3.40) |  |
| Platelet**-**PLT (x10^9^/L)  [median (IQR)] | n=90 | | **<0.0001*** | n=104 | | **<0.0001*** |
|  | 247 (158) | 336 (226) |  | 198 (157) | 334 (197) |  |
| Total bilirubin (mg/dl)  [median (IQR)] | n=86 | | 0.882 | n=101 | | 0.720 |
|  | 0.0457  (0.0835) | 0.0293  (0.0792) |  | 0.0626  (0.1003) | 0.0378  (0.1009) |  |
| Aspartate amino transferase**-**AST (U/L) (U/L)  [median (IQR)] | n=86 | | **0.007*** | n=101 | | 0.880 |
|  | 43.1235  (21.7100) | 37.2740  (15.9245) |  | 38.8300  (17.1435) | 41.5700  (17.0515) |  |
| Alanine aminotransferase **-**ALT (U/L)  [median (IQR)] | n=86 | | 0.983 | n=100 | | 0.956 |
|  | 21.7300  (15.2300) | 21.7800  (11.5675) |  | 22.2460  (11.4425) | 11.4425  (11.5575) |  |
| Creatinine**-**CREA (mg/dl)  [median (IQR)] | n=86 | | 0.214 | n=101 | | 0.785 |
|  | 0.4931  (0.2385) | 0.5178  (0.2307) |  | 0.5247  (0.2311) | 0.5304  (0.2446) |  |

n=Number of participants enrolled on day 0 and followed**-**up until day 7; *P<0.05**-**Statiscally significant (determined by using Mann**-**Whitney U test)
